# Kidney Function, Alzheimer Disease Blood Biomarkers, and Dementia Risk in Community-Dwelling Older Adults

**DOI:** 10.1212/WNL.0000000000214446

**Published:** 2025-12-03

**Authors:** Francesca Gasparini, Martina Valletta, Davide Liborio Vetrano, Giorgi Beridze, Debora Rizzuto, Amaia Calderón-Larrañaga, Claudia Fredolini, Matilda Dale, Bengt Winblad, Laura Fratiglioni, Giulia Grande

**Affiliations:** 1 Aging Research Center, Department of Neurobiology, Care Sciences and Society, Karolinska Institutet and Stockholm University, Stockholm, Sweden;; 2 Stockholm Gerontology Research Center, Stockholm, Sweden;; 3 Affinity Proteomics Stockholm, Science for Life Laboratory, Department of Protein Science, School of Engineering Sciences in Chemistry, Biotechnology and Health (CBH), Royal Institute of Technology (KTH), Solna, Sweden;; 4 Division of Neurogeriatrics, Department of Neurobiology, Care Sciences and Society, Center for Alzheimer Research, Karolinska Institutet, Solna, Sweden; and; 5 Theme Inflammation and Aging, Karolinska University Hospital, Huddinge, Sweden.

## Abstract

**Background and Objectives:**

Impaired kidney function has been linked to altered concentrations of blood biomarkers of Alzheimer disease (AD), but the underlying mechanisms and its potential role in dementia development remain poorly understood. We explored the associations between estimated glomerular filtration rate (eGFR), blood-based biomarkers of AD, and dementia development.

**Methods:**

Data were extracted from the Swedish National Study on Aging and Care in Kungsholmen, an ongoing longitudinal population-based study. Kidney function was assessed using eGFR based on serum creatinine. AD biomarkers (amyloid beta [Aβ42/40], phosphorylated tau [p-tau181 and p-tau217] and total tau [t-tau] proteins, neurofilament light chain [NfL], and glial fibrillary acidic protein [GFAP]) were measured from peripheral blood samples using the Simoa platform. Dementia was diagnosed according to DSM-IV criteria. Quantile regression models assessed the cross-sectional associations between eGFR and AD biomarkers; Cox regression models were used to examine the association of kidney function and biomarkers with incident dementia.

**Results:**

At baseline, 2,279 dementia-free participants with available blood samples were included (median age 72 (interquartile range, 61–81) years; 62% female). Lower eGFR was associated with higher median z-score levels of all examined AD blood biomarkers, except Aβ42/40, following a nonlinear relationship. At eGFR = 30 mL/min/1.73 m^2^, estimated differences were as follows: p-tau181: β, 0.22 [95% CI 0.09–0.35]; p-tau217: β, 0.20 [95% CI 0.10–0.31]; t-tau: β, 0.24 [95% CI 0.05–0.42]; NfL: β, 0.88 [95% CI 0.80–0.95]; GFAP: β, 0.10 [95% CI 0.03–0.16]. During a mean follow-up period of 8.3 (SD, 4.3) years, 362 participants developed dementia. In multivariable-adjusted models, impaired kidney function (eGFR < 60 mL/min/1.73 m^2^) was not associated with an increased hazard of dementia compared with preserved kidney function (eGFR ≥ 60 mL/min/1.73 m^2^) (hazard ratio [HR], 0.93 [95% CI 0.72–1.21]). The relationship between increased (high vs low) NfL and dementia was stronger among individuals with impaired (vs preserved) kidney function (HR, 3.85 [95% CI 1.87–7.95] vs HR, 1.84 [95% CI 1.34–2.53], respectively).

**Discussion.:**

Impaired kidney function was associated with elevated circulating level of most AD blood biomarkers. However, the presence of impaired kidney function did not independently increase the risk of dementia but rather seemed to accelerate the clinical expression of underlying neurodegenerative pathology.

## Introduction

Blood-based biomarkers of Alzheimer disease (AD) have shown to accurately correlate with their CSF counterparts and with PET hallmarks of AD, and to predict cognitive decline and impending dementia.^1-6^ Owing to their less invasive nature and relatively lower cost compared with traditional AD biomarkers such as CSF analysis and PET imaging, these biomarkers hold significant potential for entering into clinical practice soon.^3^

However, blood-based biomarkers' bioavailability can be influenced by several somatic conditions, in addition to reflecting brain pathology.^7-11^ Emerging evidence indicates that impaired kidney function could be one such factor,^7-9,11,12^ although most previous studies have focused on a limited set of biomarkers at a time. Namely, chronic kidney disease (CKD) has been consistently associated with higher levels of amyloid-β (42 and 40),^7,8^ phosphorylated tau (p-tau181)^9,11^ and total tau (t-tau)^7-9^ proteins, neurofilament light chain (NfL),^7-9^ and glial fibrillary acidic protein (GFAP)^9^ in blood. Notably, only 1 population-based study included p-tau217^11^ and found that CKD might affect the clinical interpretation of this marker, which has been recently considered as a standalone biomarker for AD diagnosis and staging.^13-15^ However, whether reduced kidney function influences the circulating concentration of biomarkers as a consequence of decreased protein clearance in the bloodstream^11,12^ or through directly contributing to brain pathology^16^ is still debated. In addition, there is currently conflicting evidence regarding the role of kidney impairment in the development of dementia,^17^ with some studies suggesting an increased risk,^16,18-20^ while others report no such association.^12,21,22^ Finally, literature exploring whether kidney function modifies the association between AD blood biomarkers and dementia is currently lacking. A disease-modifying role of kidney impairment, rather than its role as a risk factor, might explain previous conflicting results on the studied association. In other words, CKD might facilitate dementia development in the presence of preexisting neuropathology rather than directly driving dementia onset. As such, there is a need to disentangle the role of kidney function in brain aging and specifically in the development of dementia in real-world settings.

The aims of this study were (1) to explore the cross-sectional association between estimated glomerular filtration rate (eGFR) and a comprehensive panel of AD blood biomarkers; (2) to quantify the hazard of dementia across different eGFR values over a 16-year period; and (3) to investigate the potential modifying effect of kidney function in the association between AD blood biomarkers and dementia.

## Methods

### Study Population

This study is based on data from the Swedish National Study on Aging and Care in Kungsholmen (SNAC-K), an ongoing longitudinal population-based study including randomly selected individuals aged 60 years or older from 11 age cohorts (60, 66, 72, 78, 81, 84, 87, 90, 93, 96, and ≥99 years) living in the Kungsholmen district in central Stockholm.^23^ Of the 5,111 individuals invited, 4,590 were eligible and 3,363 (73.3%) completed the baseline assessment between 2001 and 2004. Follow-up evaluations were conducted every 6 (participants aged <78 years) or 3 (participants aged ≥78) years. For this study, the analytical sample consisted of dementia-free participants with available measures of AD blood biomarkers and serum creatinine at baseline.

### Standard Protocol Approvals, Registrations, and Participant Consents

Written informed consent was gathered from participants or their close relatives for those with cognitive impairment. All SNAC-K protocols received approval from the Regional Ethical Review Board in Stockholm, Sweden. Results are reported in keeping with the Strengthening the Reporting of Observational Studies in Epidemiology recommendations.

### Data Collection

At baseline and at each follow-up visit, participants underwent a comprehensive assessment by trained staff, which included medical examinations, interviews conducted by nurses, cognitive evaluations administered by psychologists, and laboratory tests completed on the same day. For blood sample collection, fasting was not compulsory and 95% (N = 2,166) were not fasting. Demographic information was collected through face-to-face interviews with the nursing staff. Participants' educational attainment was categorized into elementary, high school, or university and above. DNA for *APOE* genotyping was derived from peripheral blood samples; participants were categorized as *APOE* ε4 carriers if they had at least 1 *APOE* ε4 allele and as noncarriers if they did not have any. Body mass index (BMI) was calculated as weight in kilograms divided by height in meters squared.

### Kidney Function

Serum creatinine was measured from peripheral blood samples provided by the participants at each visit. Creatinine measurements performed before standardization to isotope dilution mass spectrometry were reduced by 5% as suggested in previous studies.^24^ We calculated eGFR using the 2009 Chronic Kidney Disease-Epidemiology Collaboration (CKD-EPI) equation based on serum creatinine.^25^ According to the Kidney Disease: Improving Global Outcomes guidelines,^26^ kidney function was considered impaired when eGFR was below 60 mL/min/1.73 m^2^.

### AD Blood Biomarkers

AD biomarkers were obtained from peripheral blood samples provided by the participants at baseline. We measured amyloid beta (Aβ42 and Aβ40), phosphorylated tau at residual threonine 181 and 217 (p-tau181 and p-tau217, respectively), total tau (t-tau), NfL, and GFAP. Because Aβ42/40 has been shown to exhibit higher diagnostic accuracy than Aβ42 alone,^2^ we calculated and included this ratio in the final analysis. On blood centrifugation, serum aliquots were stored at −80°C in the Karolinska Institutet Bio Bank until the time of analyses. Protein quantification was measured using ultrasensitive single-molecule array (Simoa) technology at the Affinity Proteomics Stockholm Unit (SciLifeLab). The Quanterix instruments provide relative average enzyme per bead values for calibrators, controls, and samples for each protein; Quanterix SR-X software can automatically perform curve fitting, extrapolation of concentrations, and graphical representation of the results. The Simoa Neuro 3-Plex A Kit was used for amyloid β isoforms and t-tau, Simoa p-tau181 Advantage V2 Kit for p-tau181, and Simoa Neuro 2-plex B Kit for NfL and GFAP. p-tau217 was quantified using the commercial assay Simoa ALZpath p-tau217 Advantage PLUS developed for the Quanterix HD-X system. Single-value imputation was used to replace data below the limit of detection with a value of 0; in total, we imputed 6 measures for Aβ42, 15 for both p-tau181 and t-tau, and 5 for p-tau217.

### Chronic Disease Assessment

Chronic diseases were identified through a comprehensive clinical assessment conducted by the examining physicians at each visit, as previously detailed.^27^ Diagnoses were established considering participant and proxy self-reports, results from medical examinations, laboratory and diagnostic tests, medication use, and a review of medical charts. In addition, this information was supplemented with diagnoses from inpatient and outpatient care visits obtained from the Swedish National Patient Register. All diagnoses were coded according to the International Classification of Diseases, 10th Revision (ICD-10). In this study, cardiovascular and cerebrovascular diseases included heart failure, ischemic heart disease, atrial fibrillation, and stroke or TIA.

### Dementia Diagnosis

A clinical diagnosis of dementia, in keeping with the Diagnostic and Statistical Manual of Mental Disorders, Fourth Edition (DSM-IV), criteria,^28^ was consistently applied across all waves using a three-step procedure. The diagnostic process was based on a comprehensive evaluation that included a detailed medical history and a general and neurologic examination. Cognitive function was assessed using structured questions addressing problem solving, abstract reasoning, orientation, and general knowledge. Standardized tools included the Mini-Mental State Examination, the clock-drawing test, digit span tasks, and a short story assessment of frontal lobe function. Functional independence in both basic and instrumental activities of daily living was also systematically evaluated. The initial diagnosis was conducted by the examining physician, followed by a second diagnosis by a reviewing physician. In instances of disagreement between these 2 assessments, a neurologist external to the data collection process determined the final diagnosis. For participants who died between 2 visits without a dementia diagnosis, additional information was gathered through (1) the clinical charts and medical records of the deceased participant and (2) causes of death registered in the Swedish National Cause of Death Register.

### Statistical Analyses

Sociodemographic and clinical characteristics of participants were reported as median and interquartile range (IQR) and frequencies (percentages) for continuous and categorical variables, respectively. To facilitate accurate comparisons among AD blood biomarkers, standardization into average z-scores using baseline mean and SD was conducted.

### Association Between Kidney Function and AD Blood Biomarkers

Quantile regression on the median was used to assess the relationship between the standardized concentrations of AD blood biomarkers and eGFR, which was modeled using restricted cubic spline with 3 prespecified knots at 30, 60, and 90 mL/min/1.73 m^2^. These values were chosen a priori based on literature review as they reflect clinically meaningful thresholds for CKD prognosis.^26^ Multivariable analyses were conducted with age, sex, education, *APOE* ε4 carrier status, anemia (present vs absent), any cardiovascular and cerebrovascular disease (defined as at least one among atrial fibrillation, heart failure, ischemic heart disease, and stroke/TIA), and BMI as covariates. Because creatinine-based equations may overestimate true GFR in individuals with low muscle mass or sarcopenia, we conducted supplementary analyses adjusting for calf circumference instead of BMI. The Wald test was used to test for statistical significance. To test whether these results were influenced by impending dementia, we repeated the analyses excluding those individuals who developed dementia during the entire 16-year follow-up period (n = 362). This approach allowed us to minimize the potential influence of prodromal dementia in the studied association.

### Association Between Kidney Function and Incident Dementia

The Cox regression model was used to investigate the association between kidney function at baseline (preserved and impaired kidney functions refer to eGFR ≥ or <60 mL/min/1.73 m^2^, respectively) and incident dementia over a 16-year period. Multivariable analyses were conducted using the same covariates as in the quantile regression models, and adjusted hazard ratios (HRs) with 95% CI were obtained. Follow-up time was defined as the time (measured in years) between baseline visit and all-cause dementia diagnosis, loss to follow-up, or death. On average, participants have been assessed 2 times, ranging from 1 to 5. To account for the possible effect of the evolution of eGFR and chronic conditions over the follow-up time, we conducted further analyses by entering eGFR (modeled using restricted cubic spline), age, and other covariates into the model as time-varying variables. The proportional hazard assumption was tested with Schoenfeld residuals, and no deviation was detected.

### Role of Kidney Function in the Association Between AD Blood Biomarkers and Incident Dementia

Finally, we tested for multiplicative interactions between each biomarker and kidney function and conducted stratified analyses using Cox regression models to assess whether the association between AD blood biomarkers and incident dementia varied by kidney function. To facilitate clinical interpretation, for this set of analyses, biomarkers were dichotomized (low and high levels) using cutoff values derived from a nonparametric bootstrapping method that maximized Youden index for 10-year all-cause dementia, as previously reported by our group.^1^ To account for the potential loss of information resulting from dichotomization, we conducted sensitivity analyses modeling biomarkers as continuous variables.

Data were analyzed and visualized using STATA version 17.0 (StataCorp LLC, College Station, TX). Significance level was set at α = 0.05. STATA and GraphPad Prism 9 were used for graphical representations.

### Data Availability

Data were derived from the SNAC-K project, a population-based study on aging and dementia (snac-k.se/). Access to the original data is available to the research community on approval by the SNAC-K data management and maintenance committee. Applications for accessing the data can be submitted to snac-k.se/.

## Results

### Characteristics of the Study Population

Of the 3,363 participants attending the baseline visit between 2001 and 2004, we excluded 240 individuals with prevalent dementia and 10 individuals with missing information on dementia status, leaving a sample of 3,113 dementia-free people. Of those, 665 individuals did not have available blood samples and 169 individuals had missing measurements of any AD blood biomarkers or serum creatinine. The study flowchart (eFigure 1) illustrates the participant selection process for the analyses. Compared with the study sample (N = 2,279), the 834 participants without available measurements for either creatinine or biomarkers were older (72 vs 78 years), were less educated (36% vs 29% attained university education), and had a slightly higher burden of chronic diseases (on average 3 vs 4 comorbidities).

Baseline characteristics of the study population overall and by kidney function are summarized in Table 1. The median age of the 2,279 participants was 72 years (IQR, 61–81), 62% were female, 36% attained university education, and 29% were carriers of at least 1 *APOE* ε4 allele.

**Table 1:** Baseline Characteristics of the Study Sample Overall and by Kidney Function

	Total	Preserved kidney function	Impaired kidney function	*p* Value
	(N = 2,279)	(N = 1,722)	(N = 557)	
Age, y	72 (61–81)	66 (61–73)	82 (78–90)	<0.001
Sex (female), %	1,405 (62)	997 (58)	408 (73)	<0.001
Education, %				<0.001
Elementary school	337 (15)	215 (12)	122 (22)	
High school	1,116 (49)	803 (47)	313 (56)	
University	825 (36)	703 (41)	122 (22)	
*APOE* ε4 carrier	652 (29)	518 (30)	134 (24)	
BMI, kg/m^2^	25 (23–28)	26 (23–28)	25 (22–28)	0.014
Comorbidities, %				
Anemia	237 (10)	113 (7)	124 (22)	<0.001
Atrial fibrillation	189 (8)	103 (6)	86 (15)	<0.001
Heart failure	176 (8)	64 (4)	112 (20)	<0.001
Ischemic heart disease	305 (13)	176 (10)	129 (23)	<0.001
Cerebrovascular disease	131 (6)	83 (5)	48 (9)	<0.001
Kidney function				
Creatinine, mg/dL	0.92 (0.83–1.03)	0.87 (0.81–0.97)	1.10 (0.99–1.27)	<0.001
eGFR, mL/min/1.73 m^2^	70 (60–79)	74 (68–82)	51 (44–56)	<0.001
Blood biomarkers				
Aβ42/40	0.06 (0.05–0.07)	0.06 (0.05–0.07)	0.05 (0.05–0.06)	<0.001
p-tau181, pg/mL	1.18 (0.74–1.79)	1.04 (0.69–1.56)	1.75 (1.16–2.53)	<0.001
p-tau217, pg/mL	0.10 (0.06–0.18)	0.09 (0.05–0.14)	0.17 (0.10–0.29)	<0.001
t-tau, pg/mL	0.84 (0.55–1.18)	0.78 (0.50–1.10)	1.03 (0.73–1.45)	<0.001
NfL, pg/mL	17.98 (12.52–28.34)	15.44 (11.48–21.98)	33.72 (23.51–49.91)	<0.001
GFAP, pg/mL	121.01 (79.99–188.41)	105.35 (72.59–152.08)	198.63 (136.09–290.98)	<0.001

Abbreviations: Aβ = amyloid beta; BMI = body mass index; eGFR = estimated glomerular filtration rate; GFAP = glial fibrillary acidic protein; NfL = neurofilament light chain; p-tau181 = phosphorylated tau 181; p-tau217 = phosphorylated tau 217; t-tau = total tau.

Data are presented as median (IQR) for continuous measures and n (%) for categorical measures.

*p* Values are derived from the Mann-Whitney test and χ^2^ test as appropriate. Missing data included 1 participant for education and 68 for *APOE* ε4 carrier status.

Preserved and impaired kidney functions refer to eGFR ≥ and <60 mL/min/1.73 m^2^, respectively.

Overall, 557 participants (24%) had impaired kidney function (eGFR < 60 mL/min/1.73 m^2^); these individuals were older and mostly female and had a higher comorbidity burden, compared with participants with preserved kidney function.

For longitudinal analyses, we used data from baseline to wave 6 (2016–2019), resulting in a maximum follow-up of 16 years. A total of 142 participants withdrew from the study after baseline assessment; these individuals were younger (61 vs 72 years), were more educated (29% vs 27% attained university education), and had fewer comorbidities (2 vs 3) compared with those who remained in the study.

### Kidney Function and AD Blood Biomarkers

**Figure 1:**
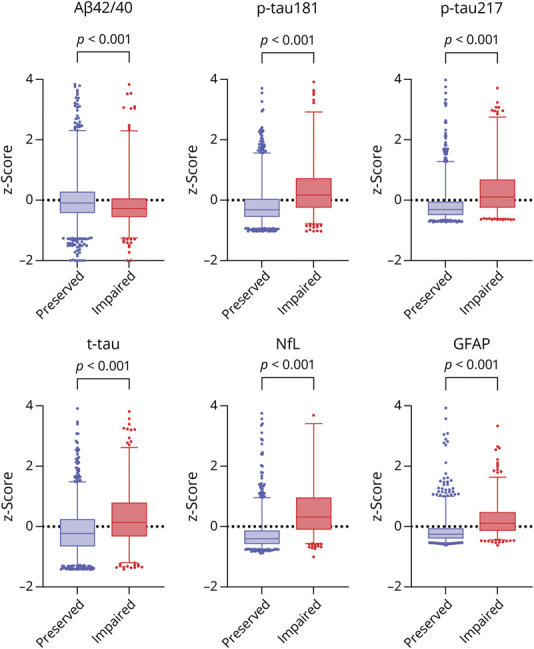
Distribution of Blood-Based Biomarkers of Alzheimer Audio Volume Mute Disease (z-Scores) by Kidney Function Box plots show the median (central line) and interquartile range (box) as well as the 2.5th and 97.5th percentiles (whiskers). *p* values are derived from the Mann-Whitney test. Values above 4 SD and below −2 SD have been excluded from the graph for graphical purposes. Preserved and impaired kidney functions refer to eGFR ≥ and <60 mL/min/1.73 m^2^, respectively. Aβ = amyloid beta; eGFR = estimated glomerular filtration rate; GFAP = glial fibrillary acidic protein; NfL = neurofilament light chain; p-tau181 = phosphorylated tau 181; p-tau217 = phosphorylated tau 217; t-tau = total tau.

At baseline, individuals with impaired kidney function had higher concentrations of tau proteins, NfL, and GFAP and lower levels of Aβ42/40 compared with participants with an eGFR ≥ 60 mL/min/1.73 m^2^ (*p* < 0.001 for all) (Table 1, Figure 1).

**Figure 2:**
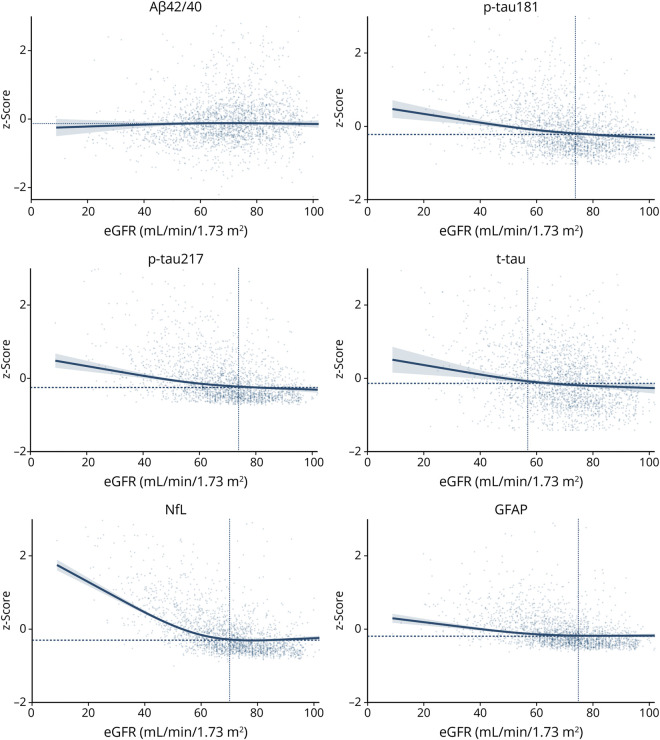
Association Between Estimated Glomerular Filtration Rate and Blood-Based Biomarkers of Alzheimer's Disease (z-Scores) Estimates are derived from quantile regression models on the median adjusted for age, sex, education, *APOE* ε4 carrier status, anemia, any cardiovascular or cerebrovascular disease (defined as at least 1 among atrial fibrillation, heart failure, ischemic heart disease, and stroke/TIA), and body mass index. Values of standardized biomarkers above 3 and of eGFR above 100 mL/min/1.73 m^2^ have been excluded from the graph. Estimated GFR is modeled using restricted cubic spline function with 3 prespecified knots (at 30, 60, and 90 mL/min/1.73 m^2^ of eGFR). The horizontal dashed line refers to the standardized median concentration of blood-based biomarkers, corresponding to −0.13 for Aβ42/40, −0.22 for p-tau181, −0.25 for p-tau217, −0.14 for t-tau, −0.30 for NfL, and −0.19 for GFAP. The vertical dotted line refers to the threshold of eGFR below which we could detect a significant increase in the standardized concentration of blood-based biomarkers, corresponding to 74 mL/min/1.73 m^2^ for p-tau181, 74 mL/min/1.73 m^2^ for p-tau217, 57 mL/min/1.73 m^2^ for t-tau, 70 mL/min/1.73 m^2^ for NfL, and 75 mL/min/1.73 m^2^ for GFAP. Aβ = amyloid beta; eGFR = estimated glomerular filtration rate; GFAP = glial fibrillary acidic protein; NfL = neurofilament light chain; p-tau181 = phosphorylated tau 181; p-tau217 = phosphorylated tau 217; t-tau = total tau.

Multivariable quantile regression analyses showed that lower eGFR was associated with higher median levels of t-tau, p-tau181 and p-tau217, NfL, and GFAP, following a nonlinear relationship (*p* for nonlinearity≤0.001 for all) (Figure 2), with NfL exhibiting the strongest association with reduced kidney function. For instance, the concentration of NfL increased by approximately 1 SD at 30 mL/min/1.73 m^2^ of eGFR (β = 0.88 [95% CI 0.80–0.95]) while the other biomarkers presented smaller increases (t-tau181: β, 0.22 [95% CI 0.09–0.35]; t-tau217: β, 0.20 [95% CI 0.10–0.31]; t-tau: β. 0.24 [95% CI 0.05–0.42]; GFAP: β, 0.10 [95% CI 0.03–0.16]).

A nonlinear relationship was also observed between lower eGFR and Aβ42 and Aβ40 individually (eFigure 2) while no significant association emerged with their ratio (Aβ42/40, *p* for nonlinearity = 0.6321).

After excluding those individuals who developed dementia during the 16-year follow-up period (N = 362), results remained largely unchanged (eFigure 3). Similar results were also observed when repeating the analysis adjusting for calf circumference instead of BMI (eFigure 4).

### Kidney Function and the Hazard of Dementia

During a mean of 8.3 (SD, 4.3) years of follow-up, 362 dementia cases were detected including 221 among participants with preserved kidney function and 141 among those with impaired kidney function (unadjusted incidence rates of dementia per 100 person years, 1.46 (95% CI 1.28–1.67) and 3.72 (95% CI 3.16–4.39), respectively). Characteristics of participants by dementia status are provided in eTable 1.

Compared with participants with preserved kidney function, and independently from confounders, those with impaired kidney function did not show an increased hazard of dementia (HR, 0.93 [95% CI 0.72–1.21]).

**Figure 3:**
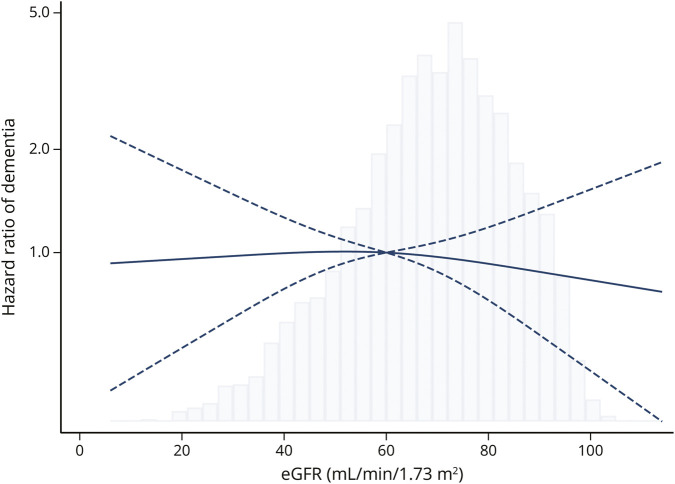
Association Between Estimated Glomerular Filtration Rate and Dementia Estimates are HRs derived from the Cox proportional hazard regression model adjusted for age, sex, education, *APOE* ε4 carrier status, anemia, any cardiovascular or cerebrovascular disease (defined as at least 1 among atrial fibrillation, heart failure, ischemic heart disease, and stroke/TIA), and body mass index. The following variables have been included as time-varying covariates: age, eGFR, anemia, any cardiovascular or cerebrovascular disease, and body mass index. Estimated GFR is modeled using restricted cubic spline function with 3 prespecified knots (at 30, 60, and 90 mL/min/1.73m2 of eGFR); the reference group corresponds to 60 mL/min/1.73 m2 of eGFR. Values of eGFR above 100 mL/min/1.73m^2^ have been excluded from the graph. The solid line indicates point estimates; the dashed line the 95% CI. The histogram in the background represents the distribution of eGFR in the study sample. eGFR = estimated glomerular filtration rate.

Similar results were observed when exploring the association between eGFR and dementia, considering both exposure and covariates as time-changing variables. No higher hazard of dementia was observed in relation to more severe kidney impairment (i.e., HR, 1.00 [95% CI 0.85–1.18] at 45 mL/min/1.73 m^2^; HR, 0.98 [95% CI 0.65–1.48] at 30 mL/min/1.73 m^2^) compared with the reference group (eGFR = 60 mL/min/1.73 m^2^) (Figure 3).

### Role of Kidney Function in the Association Between AD Blood Biomarkers and Incident Dementia

**Figure 4:**
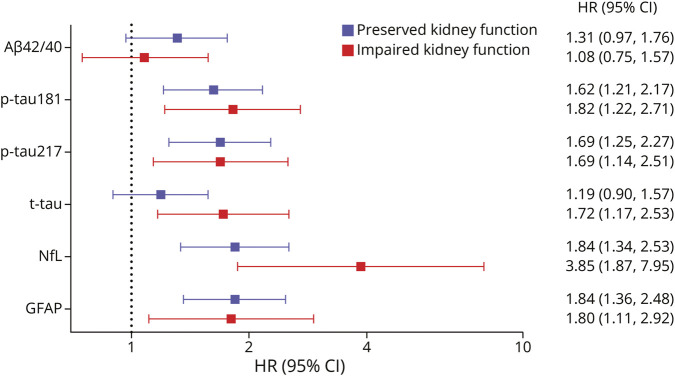
Association Between Blood-Based Biomarkers of Alzheimer Disease and Incident Dementia, According to Kidney Function Estimates are hazard ratios (HRs) of dementia with 95% CIs derived from stratified Cox proportional hazard regression models exploring the association between high (vs low) levels of biomarkers and incident dementia including interaction terms between biomarkers and kidney function, adjusted for age, sex, education, *APOE* ε4 carrier status, anemia, any cardiovascular or cerebrovascular disease (defined as at least 1 among atrial fibrillation, heart failure, ischemic heart disease, and stroke/TIA), and body mass index. For Aβ42/40, high (vs low) level was chosen as the reference. Biomarkers were dichotomized (low and high levels) using the following cutoff values previously reported by our group^1^: 0.057 for Aß42/40, 0.134 pg/mL for p-tau217, 1.512 pg/mL for p-tau181, 0.832 pg/mL for t-tau, 20.171 pg/mL for NfL, and 142.515 pg/mL for GFAP. Preserved and impaired kidney functions refer to eGFR ≥ and <60 mL/min/1.73 m^2^, respectively. *p* for interaction: Aβ42/40#kidney function: *p* = 0.433; p-tau181#kidney function: *p* = 0.649; p-tau217#kidney function: *p* = 0.991; t-tau#kidney function: *p* = 0.128; NfL#kidney function: *p* = 0.064; GFAP#kidney function: *p* = 0.944. Aβ = amyloid beta; eGFR = estimated glomerular filtration rate; GFAP = glial fibrillary acidic protein; NfL = neurofilament light chain; p-tau181 = phosphorylated tau 181; p-tau217 = phosphorylated tau 217; t-tau = total tau.

Figure 4 shows the relationship between AD blood biomarkers and dementia risk, stratified by kidney function. Incidence rates of dementia by AD pathology and kidney function are provided in eTable 2. No statistically significant interaction was detected between kidney function and categories of biomarkers for dementia development. However, stratified analyses indicated that the association between high levels of NfL and dementia was stronger among individuals with impaired kidney function (HR, 3.85 [95% CI 1.87–7.95]) compared with those with preserved kidney function (HR, 1.84 [85% CI, 1.34–2.53]). A significant association between increased levels of t-tau and dementia was observed only among individuals with impaired kidney function (HR, 1.72 [95% CI 1.17–2.53]), whereas no significant association was found in those with eGFR ≥ 60 mL/min/1.73 m^2^ (HR, 1.19 [95% CI 0.90–1.57]). The association of p-tau181, p-tau217, and GFAP with dementia was similar in individuals with and without impaired kidney function, whereas Aβ42/40 was not significantly associated with increased dementia risk in either group.

Attenuated but consistent results were observed for p-tau181, p-tau217, and NfL when biomarkers were treated as continuous variables in sensitivity analyses (eTable 3), with evidence of interaction between NfL and kidney function (impaired kidney function: HR, 1.47 [95% CI 1.26, 1.70] vs preserved kidney function: HR, 1.14 [95% CI 1.03, 1.26]; p for interaction = 0.005). However, no significant associations emerged for t-tau and GFAP in this set of analysis.

## Discussion

This study, performed in a large community-dwelling population of cognitively unimpaired older adults, yielded 3 main findings. First, impaired kidney function was linked to greater concentrations of all AD blood biomarkers, except for Aβ42/40, with the strongest association observed for NfL. Of note, results were confirmed even after restricting the analysis to those individuals who never developed dementia over the entire 16-year follow-up period. Second, impaired kidney function was not independently associated with faster development of dementia, and this finding was further confirmed when a time-varying approach for eGFR and comorbidities was used. Third, kidney impairment seemed to accelerate the development of dementia among participants with evidence of neurodegenerative processes, as described by increased levels of NfL.

Our cross-sectional findings are in line with previous population-based studies, where AD blood biomarkers—particularly NfL—were shown to be altered in the presence of impaired kidney function.^7-9,11,12,18,29-31^ In fact, 1 previous study from our group^9^ and 2 studies from the Mayo Clinic Study of Aging^7,11^ found that CKD was one of the strongest conditions associated with altered levels of p-tau,^9,11^ t-tau,^7,9^ NfL,^7,9^ and GFAP^9^ in blood. We not only confirmed but also expanded previous knowledge, as we explored an extensive panel of AD blood biomarkers, including p-tau217, which was only considered in one previous cross-sectional population-based study where CKD was derived from medical records.^11^ The study of the factors that may alter p-tau217 levels in the blood is essential, as this biomarker has been recently defined as a standalone biomarker for AD diagnosis and staging.^13^ Of interest, the difference in plasma p-tau levels between participants with and without CKD was similar to that observed between participants with and without elevated brain amyloid in one previous study.^11^ Accordingly, the association between eGFR and both p-tau isoforms remained largely unchanged after restricting the analyses to those individuals who did not develop dementia during the follow-up in our study, reinforcing the hypothesis that the observed relationship was not driven by prodromal dementia. In line with previous knowledge,^8^ the association of impaired kidney function was particularly evident on NfL, which is a nonspecific biomarker of neurodegeneration.^3,32^ Furthermore, the authors of a prior study^12^ observed no significant association between eGFR groups and GFAP. Similarly, the levels of GFAP were rather stable across the range of eGFR in our study, thus hypothesizing that this marker is less affected by peripheral factors compared with the others. Finally, both Aβ42 and Aβ40 seemed to be influenced by kidney impairment,^8^ although we did not observe an association when considering the ratio of the 2 biomarkers instead—which is in line with previous works.^8,9,18^ Similarly, a previous study^33^ found that plasma ratios of phosphorylated-to-unphosphorylated tau were less strongly associated with CKD than p-tau forms alone. As such, implementing biomarker ratios might reduce comorbidity-related confounding and provide more accurate measures of brain pathology. Notably, in the Alzheimer's Association Clinical Practice Guideline,^34^ blood-based biomarkers should be used in a comprehensive clinical evaluation and only as part of a full diagnostic workup of patients with cognitive impairment.

The possible mechanisms by which kidney dysfunction alters the circulating levels of AD blood biomarkers are not yet fully understood—and include peripheral mechanisms, direct effects on neuropathology, or both. Indeed, it is plausible to hypothesize that reduced GFR impairs the peripheral clearance of these molecules, subsequently leading to an increase in their blood levels.^35^ Nonetheless, it is also plausible that a decline in kidney function is directly implicated in neuropathology,^16,18^ and that biomarker levels increase as a mere consequence of it. In fact, both the accumulation of uremic toxins and the release of neuroinflammatory cytokines that accompany CKD have been associated with neurotoxicity and nonspecific brain damage,^17,36,37^ which could contribute to dementia development. Of note, the older age and the higher comorbidity burden among participants with impaired kidney function might indicate the presence of underlying mixed brain pathology, which could also partially account for the elevated levels of biomarkers—especially NfL—observed in this subgroup.

However, we did not observe an independently higher dementia risk in individuals with reduced eGFR. In line with our findings, 3 population-based studies found no association between impaired kidney function and increased dementia risk.^12,21,22^ Consistent with the US Adult Changes in Thought study,^22^ our results were further confirmed when a time-varying approach accounting for the possible effect of kidney function decline over time was used. One possible explanation is that reduced GFR alone may not represent a pathologic feature per se among older individuals. In fact, a slow decline in GFR is physiologically expected as part of the natural aging process, regardless of the presence of renal structural or functional abnormalities.^38^

To further explore the intricate mechanisms underlying the kidney-brain axis, we evaluated whether kidney function modified the association between AD pathology, as measured using biomarkers, and future dementia. We observed that the dementia risk associated with NfL—and, to a lesser extent, t-tau—was higher among individuals with impaired kidney function. This finding indicates a possible interplay between kidney dysfunction and biomarkers of neurodegeneration, suggesting that kidney impairment may accelerate the clinical expression of underlying neurodegenerative pathology. Of note, this hypothesis was further supported by the presence of interaction between NfL and kidney function when biomarkers were treated as a continuous variable in our sensitivity analyses. Given the mutual crosstalk between CKD, vascular disease, and neurodegeneration,^17,19^ it is not surprising that the association between NfL—which is again a nonspecific biomarker of axonal and neurovascular degeneration^32^—and dementia was strongly amplified by impaired kidney function. Notably, results were also corroborated by the evidence of an increased rate of dementia onset associated with t-tau levels exclusively among participants with eGFR < 60 mL/min/1.73 m^2^. Conversely, the association of p-tau and GFAP with dementia was similar among individuals with and without impaired kidney function, suggesting that CKD might not alter the association between AD-like neuropathology and dementia development. Regarding the lack of association between Aβ42/40 and incident dementia, some biological limitations should be considered. Indeed, amyloid levels in blood are often lower than in CSF^39^ and are partly derived from peripheral sources,^40^ thus only partially reflecting brain pathology.

As such, it is plausible to hypothesize that kidney dysfunction represents a proxy of clinical complexity rather than a biological substrate independently associated with the onset of AD-related neuropathology. For instance, individuals with CKD are more prone to suffer from vascular disease, which detrimentally affects endothelial integrity and promotes vascular damage and neurodegeneration in the CNS.^17,19^ In line with this hypothesis, 1 study from the Rush Memory and Aging Project observed that the connection between impaired kidney function and dementia was mainly explained by vascular lesions.^20^ Taken together, these and previous results suggest that impaired kidney function does not act as a direct risk factor of dementia onset but may serve as a promoter of dementia development among individuals already exhibiting evidence of neurodegeneration.

These findings come from a large population-based sample of cognitively unimpaired older adults, including extensive in-person evaluations of comorbidities and clinical diagnosis of dementia. While previous studies have dichotomized kidney function or have included only a limited set of biomarkers, we were able to explore a wide spectrum of eGFR and an extensive panel of all major blood-based biomarkers of AD—including p-tau217. Both creatinine and biomarkers were measured at a single laboratory, thus minimizing between-laboratory variations in the values. These aspects, together with the opportunity of assessing repeated estimates of GFR in our longitudinal analyses, are the major strengths of this study. In addition, the extensive review of clinical records and death registers allowed us to comprehensively identify dementia cases among those who died during follow-up, thereby improving case ascertainment and reducing potential outcome misclassification.

Some limitations should also be mentioned. First, AD blood biomarkers were measured at a single time point; therefore, we cannot evaluate whether changes in kidney function might affect their concentrations longitudinally. Second, creatinine-based equations might overestimate true GFR in individuals with low muscle mass or sarcopenia. However, we attempted to mitigate this bias by including eGFR as a continuous measure instead of categorizing it into stages of kidney function and by conducting supplementary analyses adjusting for calf circumference, observing similar results. Third, our longitudinal findings should be interpreted with caution. In fact, the paucity of dementia cases and the low number of participants with severely impaired renal function in our sample could account for the estimates being less precise and prevent us from detecting a statistically significant association between lower eGFR and higher risk of dementia. Future studies are needed to better evaluate the pathophysiologic role of kidney dysfunction in the studied associations and to assess the extent to which it affects the clinical validity of AD blood biomarkers. Finally, SNAC-K consists predominantly of highly educated Caucasians from an urban setting, which may limit the generalizability of our findings to more diverse populations. Future studies including participants from a broader range of racial and ethnic backgrounds are needed to validate and extend our results.

In this large, population-based cohort of dementia-free older adults, impaired kidney function was associated with higher circulating levels of all examined AD blood biomarkers, except Aβ42/40. However, the presence of impaired kidney function did not independently increase the risk of dementia over the subsequent 16 years, but rather seemed to accelerate dementia onset among individuals with altered levels of biomarkers linked to neurodegeneration. These findings suggest that kidney impairment may act as a modifier and booster of disease expression rather than a risk factor per se, potentially influencing the timing of clinical dementia onset in the presence of brain pathology.

## Glossary

AD: Alzheimer disease
BMI: body mass index
CKD: chronic kidney disease
eGFR: estimated glomerular filtration rate
GFAP: glial fibrillary acidic protein
HR: hazard ratio
IQR: interquartile range
NfL: neurofilament light chain
SNAC-K: Swedish National Study on Aging and Care in Kungsholmen
